# Di-phosphorylated BAF shows altered structural dynamics and binding to DNA, but interacts with its nuclear envelope partners

**DOI:** 10.1093/nar/gkab184

**Published:** 2021-03-21

**Authors:** Agathe Marcelot, Ambre Petitalot, Virginie Ropars, Marie-Hélène Le Du, Camille Samson, Stevens Dubois, Guillaume Hoffmann, Simona Miron, Philippe Cuniasse, Jose Antonio Marquez, Robert Thai, François-Xavier Theillet, Sophie Zinn-Justin

**Affiliations:** Institute for Integrative Biology of the Cell (I2BC), CEA, CNRS, Université Paris-Sud, Université Paris-Saclay, Gif-sur-Yvette Cedex, France; Institute for Integrative Biology of the Cell (I2BC), CEA, CNRS, Université Paris-Sud, Université Paris-Saclay, Gif-sur-Yvette Cedex, France; Institute for Integrative Biology of the Cell (I2BC), CEA, CNRS, Université Paris-Sud, Université Paris-Saclay, Gif-sur-Yvette Cedex, France; Institute for Integrative Biology of the Cell (I2BC), CEA, CNRS, Université Paris-Sud, Université Paris-Saclay, Gif-sur-Yvette Cedex, France; Institute for Integrative Biology of the Cell (I2BC), CEA, CNRS, Université Paris-Sud, Université Paris-Saclay, Gif-sur-Yvette Cedex, France; SIMOPRO, CEA, Gif-sur-Yvette Cedex, France; High Throughput Crystallization Lab, EMBL Grenoble Outstation, Grenoble Cedex, France; Institute for Integrative Biology of the Cell (I2BC), CEA, CNRS, Université Paris-Sud, Université Paris-Saclay, Gif-sur-Yvette Cedex, France; Institute for Integrative Biology of the Cell (I2BC), CEA, CNRS, Université Paris-Sud, Université Paris-Saclay, Gif-sur-Yvette Cedex, France; High Throughput Crystallization Lab, EMBL Grenoble Outstation, Grenoble Cedex, France; SIMOPRO, CEA, Gif-sur-Yvette Cedex, France; Institute for Integrative Biology of the Cell (I2BC), CEA, CNRS, Université Paris-Sud, Université Paris-Saclay, Gif-sur-Yvette Cedex, France; Institute for Integrative Biology of the Cell (I2BC), CEA, CNRS, Université Paris-Sud, Université Paris-Saclay, Gif-sur-Yvette Cedex, France

## Abstract

Barrier-to-autointegration factor (BAF), encoded by the *BANF1* gene, is an abundant and ubiquitously expressed metazoan protein that has multiple functions during the cell cycle. Through its ability to cross-bridge two double-stranded DNA (dsDNA), it favours chromosome compaction, participates in post-mitotic nuclear envelope reassembly and is essential for the repair of large nuclear ruptures. BAF forms a ternary complex with the nuclear envelope proteins lamin A/C and emerin, and its interaction with lamin A/C is defective in patients with recessive accelerated aging syndromes. Phosphorylation of BAF by the vaccinia-related kinase 1 (VRK1) is a key regulator of BAF localization and function. Here, we demonstrate that VRK1 successively phosphorylates BAF on Ser4 and Thr3. The crystal structures of BAF before and after phosphorylation are extremely similar. However, in solution, the extensive flexibility of the N-terminal helix α1 and loop α1α2 in BAF is strongly reduced in di-phosphorylated BAF, due to interactions between the phosphorylated residues and the positively charged C-terminal helix α6. These regions are involved in DNA and lamin A/C binding. Consistently, phosphorylation causes a 5000-fold loss of affinity for dsDNA. However, it does not impair binding to lamin A/C Igfold domain and emerin nucleoplasmic region, which leaves open the question of the regulation of these interactions.

## INTRODUCTION

Barrier-to-autointegration factor, encoded by the *BANF1* gene and here referred as BAF, is an abundant and ubiquitously expressed DNA binding protein with multiple functions important for maintaining the integrity of the cellular genome (1–3). BAF A12T variant causes Nestor-Guillermo Progeria Syndrome (NGPS), a premature aging condition with early onset (4,5). BAF is highly conserved among metazoans, and BAF depletion is lethal during embryogenesis in *Caenorhabditis elegans* and *Drosophila* melanogaster (6–8). It was first identified as a host protein that captures viral DNA (1,9–11). It binds genomic self-DNA upon loss of nuclear membrane integrity, thus outcompeting cGAS and preventing aberrant immune response (12,13). However, BAF was also described as playing several essential roles during the cell cycle of metazoan cells. In *Drosophila*, it is necessary for proper centromere assembly and accurate chromosome segregation (14). At the end of mitosis, it contributes to the compaction of chromosomes (15). It is localized around the mitotic chromosomes (16), forming a dense cross-bridged chromatin that establishes a mechanical barrier and restricts nuclear membrane at the surface of chromatin. It contributes to the sealing of the nuclear envelope by recruiting LEM-domain proteins (17) anchored at the inner nuclear membrane (18,19). BAF is also involved in the repair of large nuclear envelope ruptures (20–22). Cytoplasmic BAF localizes to the rupture site by binding to the leaking DNA, and recruits LEM-domain proteins anchored at the inner nuclear membrane, in order to trigger nuclear envelope repair and restore the nucleocytoplasmic barrier.

**Figure 1.**
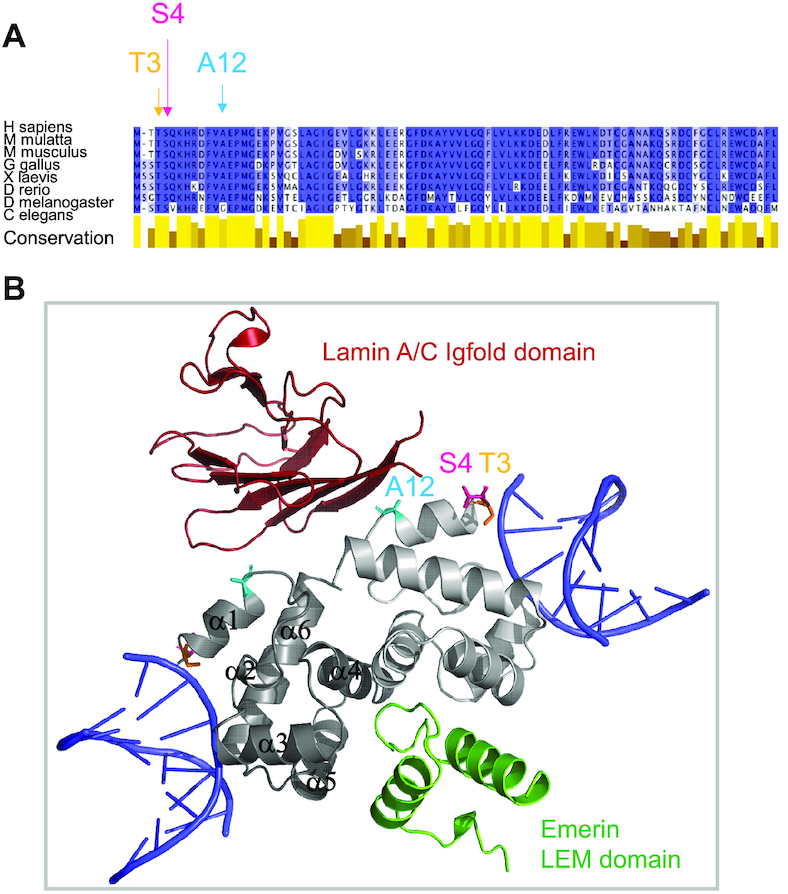
BAF is a conserved protein, binding double-stranded DNA, lamin A/C and emerin. (**A**) Alignment of the sequence of human BAF with that of eight homologs, highlighting the strong conservation of BAF across metazoans. Positions discussed in this study are marked with arrows. The conservation score was plotted using Jalview 2.10.1. (**B**) Cartoon representation of BAF bound to two double-stranded DNA, the Igfold domain of lamin A/C and the LEM domain of emerin. This model was produced by superimposing the structure of BAF in a BAF-dsDNA complex (PDB code 2BZF, the BAF monomers being in light and dark grey, respectively, and the two dsDNA in blue) with that of our BAF construct (the cysteines being mutated into alanines) in a BAF-lamin A/C-emerin complex (PDB code: 6GHD, the lamin Igfold and the emerin LEM domains being colored in red and green, respectively). Positions discussed in this study are indicated as in (A).

At the molecular level, BAF is a 89-aa protein highly conserved from mammals to lower eukaryotes as *D. melanogaster* and *C. elegans* (Figure 1A). It binds double-stranded DNA (dsDNA) without sequence specificity (6), making contacts with the phosphate backbone in the minor groove (23). As an obligate dimer, it is able to cross-bridge two dsDNA (Figure 1B; (23,24)), thus contributing to chromatin compaction (25). BAF also interacts with several nuclear envelope proteins: it directly binds to the LEM domain of inner nuclear membrane proteins as emerin (18,26) and LAP2 (27,28); it also mediates the interaction between the LEM domain of emerin and the IgFold domain of lamin A/C (Figure 1B; (29)). Thus, BAF is able to bridge the inner nuclear membrane and the nucleoskeleton formed by lamins. All these interactions underlie the localization and functions of BAF. During interphase, BAF is predominantly found at the inner nuclear envelope (30), and this localization is enhanced by expression of lamin A/C (16). However, BAF is dynamic and mobile, in stark contrast to its nuclear envelope partners (30). As cells enter mitosis, BAF assumes a diffuse localization. Then, during late anaphase, it localizes at the core region of chromosomes (18,30). BAF and emerin become relatively immobile at the surface of chromosomes (30). Depletion of emerin increases the life time of BAF at the core region, whereas depletion of lamin A/C shortens its duration, suggesting that lamin A/C stabilizes the core structure (16). Also, in primary cell lines, BAF is mostly located in the nucleus of young proliferating cells, but it becomes mainly cytoplasmic in aged senescent cells (31). Finally, following the induction of an oxidative stress, BAF relocalizes from the nuclear envelope to the chromatin (32). These observations reveal that BAF function is tightly regulated in time and space, in order to preserve nuclear structure and genome integrity.

Cycles of phosphorylation and dephosphorylation control BAF function. Several groups reported that BAF is phosphorylated by Vaccinia Related Kinases (VRK) on its N-terminal serine and threonine residues (19,33–35) and is dephosphorylated by PP2A and its cofactor LEM4/ANKLE2 (36). The crystal structure of BAF bound to DNA shows that the N-terminus of BAF is engaged in DNA binding (Figure 1B; (23)). Consistently, phosphorylation of BAF N-terminal residues impairs BAF binding to dsDNA (15,33). Overexpression of VRK1 partially releases BAF from chromatin and delocalizes it from the nucleus to the cytoplasm (33). At the opposite, depletion of VRK1 impairs the release of BAF from the chromosomes during mitosis (35). It increases the number of anaphase bridges and multipolar spindles. It frequently results in aberrant nuclear envelope morphology without any defect in lamin A/C and emerin localization (35).

To understand the strong impact of mitotic phosphorylation on BAF association with chromatin and nuclear envelope proteins, we performed a comparative biophysical analysis of BAF before and after phosphorylation. We monitored BAF phosphorylation by VRK1, solved the 3D structure of phosphorylated BAF, characterized its dynamics in solution, and measured the impact of the observed structural variations on BAF interaction with DNA, but also lamin A/C and emerin.

## MATERIALS AND METHODS

### Protein expression vectors and constructs

Codon-optimized genes coding for human emerin from amino acid 1 to amino acid 187 (EmN) and from amino acid 1 to amino acid 46 (LEM), as well as human BAF, BAF T3A and BAF K72E were synthesized and cloned by Genscript, as reported in (29). All cysteines (C85, C80, C77, C67) were mutated into alanine in the BAF constructs. EmN, BAF, BAF T3A and BAF K72E were expressed using a pETM13 vector as fusion proteins including a histidine tag, a Tobacco Etch Virus Protease (TEV) cleavage site and the protein of interest. Plasmids coding for BAF variants S4E and A12T were obtained by mutagenesis using the Quikchange (Agilent) kit from the BAF expression vector. BAF_met_, a variant with the native methionine as residue 1 (instead of the glycine present in all other constructs because of the TEV cleavage site), was expressed using a pETM13 vector as a fusion protein including an N-terminal histidine tag followed by a SUMO protein and the BAF protein. A codon optimized gene coding for the IgFold domain of human lamin A/C from amino acid 411 to amino acid 566 (LamIgF) was synthesized and cloned into a pGEX vector by Genscript (29). The pET His10 TEV LIC expression vector coding for human VRK1 catalytic domain (amino acid 3–364) was a gift from John Chodera & Nicholas Levinson & Markus Seeliger (Addgene plasmid # 79684; http://n2t.net/addgene:79684; RRID:Addgene_79684; (37)).

### Protein expression and purification

All proteins were expressed in *Escherichia coli* BL21 DE3 Star, except VRK1 that was produced in *E. coli* Rosetta2. Bacteria were grown in rich medium (*lysogeny broth*, LB), or ^15^N/^13^C labeled M9 minimum medium for the NMR experiments. Media were supplemented with 100 μg/mL ampicillin or 30 μg/ml kanamycin depending on the plasmid, and with 80 μg/ml ampicillin and 20 μg/ml chloramphenicol for VRK1. Overexpression was induced at an optical density OD_600_ = 0.8 by supplementing the medium with 1 mM ITPG at 20°C overnight. Cells were harvested by centrifugation and cell pellets stored at –80°C.

EmN, LEM, BAF, BAF A12T, BAF T3A, BAF K72E, BAF_met_ were all insoluble after expression in *E. coli*, purification was performed in urea and followed by a refolding step. After sonication in lysis buffer (50 mM Tris pH 8, 300mM NaCl, 5% glycerol, 0.1% Triton X-100) and centrifugation at 50 000 g for 15 min at 4°C, the pellet was suspended in purification buffer (50 mM Tris pH 8.0, 150 mM NaCl, 8 M urea) for 20 min. Then, the sample was centrifuged again and the soluble fraction incubated on Ni-NTA beads for 30 min at room temperature. Ni-NTA beads were washed with purification buffer and the protein was directly eluted in 50 ml in the same buffer containing 1 M imidazole. Proteins were then refolded by dialysis in their respective buffer (50 mM Tris pH 8, 150 mM NaCL for BAF and LEM and 50 mM Tris pH 8.0, 30 mM NaCl, 2 mM DTT for EmN). After concentration, the histidine tag was cleaved by the TEV protease overnight at 4°C but for BAF_met_ that was cleaved using SUMO protease. Proteins were separated from the TEV protease (containing a histidine tag) by affinity chromatography, using Ni-NTA beads. Finally, a gel filtration was performed using a Superdex 200 pg HiLoad 16/600 column. The final yield was typically 0.6 mg (LB) or 0.1 mg (M9) of purified protein per liter of bacterial culture for BAF, 6 mg (LB) for LEM and 26 mg (LB) per liter for EmN.

VRK1, BAF S4E and BAF T3S4E were all soluble after expression. After sonication, the soluble extract was incubated with benzonase for 20 min at 20°C (room temperature). The lysate was then centrifuged at 50 000 g for 15 min at 4°C and loaded onto a 5 ml Ni-NTA column (FF crude, GE-Healthcare). The column was washed with purification buffer (50 mM Tris pH 8.0, 150 mM NaCL) and eluted with an imidazole gradient (0–500 mM). After concentration to 5 ml, the histidine tag was cleaved by the TEV protease during 1h30 at room temperature. Proteins were separated from the TEV protease (containing a histidine tag) by affinity chromatography, using Ni-NTA beads. VRK1 histidine tag did not impaired the phosphorylation activity of the kinase and was not removed. Finally, last contaminants were removed by gel filtration (Superdex-200 HiLoad 16/600 column). The final yield was typically 28 mg (LB) for VRK1 and 2 mg (LB) or 0.6 mg (M9) for BAF S4E of purified protein per liter of bacterial culture.

For LamIgF, after sonication at 10°C, the supernatant was incubated 20 min at room temperature with benzonase and centrifuged at 50 000 g for 15 min at 4°C. The soluble extract was then supplemented with 5mM DTT and loaded onto glutathione beads. After 1 h of incubation at 4°C, glutathione beads were washed first with 1 M NaCl buffer and purification buffer (50 mM Tris pH 7.5, 150 mM NaCl, 5 mM DTT). The GST tag was cleaved with Thrombin for 2 h. The protein was recovered in the flow-through and separated from thrombin and last contaminants using gel filtration (Superdex 200 pg HiLoad 16/600 column). The final yield was typically 20 mg (LB) or (6mg) of purified protein per liter of bacterial culture.

### Purification of phosphorylated BAF constructs

pBAF and pBAF_met_ were obtained by phosphorylation with the VRK1 purified in the lab. After the last Ni-NTA chromatography used to remove TEV protease, BAF was recovered in the flow-through. It was then supplemented and the pH was adjusted to end in the phosphorylation buffer [50 mM Tris pH 7.5, 150 mM NaCl, 5 mM ATP, 5 mM MgSO_4_, 1 mM TCEP, 1× antiproteases (Roche) and VRK1 (molar ratio versus BAF of 4%)]. After 4 h of incubation at 30°C, the phosphorylated proteins were separated from the kinase by the last step of gel filtration used in all the purification protocols. The thermal stability of pBAF was evaluated using the simplified Thermofluor assay available on the High Throughput Crystallization Laboratory (HTX Lab) of the EMBL Grenoble (38). As phosphorylation prevents BAF oligomerization, the yield was improved compared to BAF purification to reach either 1 mg (LB) or 0.3 mg (M9) per liter of bacterial culture.

### Liquid-state NMR spectroscopy

NMR experiments were performed on a 700 MHz Bruker AVANCE NEO spectrometer equipped with a triple resonance cryogenic probe. The data were processed using Topspin 4.0 (Bruker) and analyzed using CCPNMR 2.4 (39). Sodium trimethylsilylpropanesulfonate (DSS) was used as a chemical shift reference. Most experiments performed on BAF were recorded at 20°C and pH 7.2. In order to characterize each BAF variant, 2D ^1^H–^15^N HSQC spectra were acquired on a 3-mm-diameter NMR sample tube containing the 200 μM uniformly ^15^N-labeled protein, in 40 mM sodium phosphate pH 7.2, 150 mM NaCl and 95:5% H_2_O/D_2_O.

In order to assign the ^1^H, ^15^N, ^13^C backbone signals of pBAF, 3D HNCA, CBCACONH, HNCACB, HNCO and HN(CA)CO experiments were recorded at 20°C on a 4-mm-diameter NMR sample tube containing 340 μM of uniformly ^15^N/^13^C labeled pBAF in 40 mM sodium phosphate pH 7.2, 150 mM NaCl, 95:5% H_2_O:D_2_O. The same experiments were performed on a sample of 1 mM ^15^N/^13^C labeled BAF S4E, whose chemical shifts are close to that of BAF (Supplementary Figure S3B), in order to confirm previously published BAF chemical shift assignment (40).

BAF (WT and variants) phosphorylation kinetics were carried out at 30°C on a 3-mm-diameter NMR sample tube containing 100 μM (WT) or 150 μM (variants) protein in kinetics phosphorylation buffer [40 mM HEPES pH 7.2, 150 mM NaCl, 5 mM ATP, 5 mM MgSO_4_, 1 mM TCEP, 1× antiproteases (Roche), 95:5% H_2_O:D_2_O, and 150 nM of VRK1 kinase (molar ratio versus BAF of 0.1%)]. 2D ^1^H–^15^N HSQC spectra were acquired every 30 min.

^15^N R_1_, ^15^N R_2_ CPMG and ^1^H→^15^N nOe experiments were recorded at 20°C on 3 mm- and 4 mm-diameter Shigemi tubes containing 300 μM protein (BAF or pBAF) in 40 mM sodium phosphate pH 7.2, 150 mM NaCl, 95:5% H_2_O:D_2_O. ^15^N R_1_ and ^15^N R_2_ were measured using pseudo3D experiments, whereas ^1^H→^15^N nOe were measured using interleaved NONOE and NOE experiments. For BAF, ^15^N R_1_ were recorded in triplicate using from 9 to 12 time points distributed from 10 to 1500 ms, ^15^N R_2_ were recorded in quadruplet using from 11 to 12 time points distributed from 18.5 to 185 ms, and ^1^H→^15^N nOe were measured in triplicate using a relaxation delay of 6 s. For pBAF, ^15^N R_1_ were recorded in duplicate using 12 time points distributed from 10 to 1000 ms, ^15^N R_2_ were recorded in duplicate using 12 time points distributed from 18.5 to 222 ms, and ^1^H→^15^N nOe were measured in triplicate using a relaxation delay of 5 s.

To observe if the complex between LamIgF, EmN and BAF S4E or pBAF was still existing in solution, 2D ^1^H–^15^N HSQC spectra were recorded on 80 μM of ^15^N labeled LamIgF alone, with 160 μM of BAF S4E and then 80 μM of EmN, with 160 μM of pBAF and then 80 μM of EmN and with 160 μM of pBAF and then 80 μM of LEM. All these experiments were performed in 50 mM HEPES pH 7.4, 100 mM NaCl, 3-mm-diameter tubes, at 700 MHz and 20°C. A control experiment was carried out with 80 μM of ^15^N labeled LamIgF and 80 μM EmN in the same conditions. Peak intensities corresponding to assigned ^1^H–^15^N LamIgF peaks (41) were measured and compared, in order to identify peaks decreasing by >80% after addition of LamIgF partners.

#### Mass spectrometry

On-line μLC-MS was performed using an Agilent 1100 Series Binary HPLC system (Agilent Technologies, Santa Clara, CA, USA) connected to an ESI (electrospray-ion)-trap Esquire HCT mass spectrometer (Bruker-Daltonik, GmBH). As BAF and pBAF 20 μM samples contained non-volatile buffer and salts (Tris, NaCl, EDTA), they needed to be desalted prior injection into the ESI-MS system. Desalting was performed on a ProSwift RP-4H reverse phase column (Thermo-Scientific) running at 200μL/min with a linear gradient of water/MeCN/0.1%TFA from 5 to 100% MeCN/0.1% TFA in 7 min. ESI-ion trap MS was calibrated using ESI Tune Mix solution (Sigma). Spectra were acquired in ESI positive mode operated at 4.5 kV producing multiprotonated species. Multicharged spectra were then deconvoluted into averaged masses with the DataAnalysis software (Bruker-Daltonik, GmBH).

Linear mode MALDI-TOF MS was performed using a 4800 MALDI-TOFTOF mass spectrometer (AB-SCIEX). BAF and pBAF samples were spotted on MALDI plate at 2–5 μM (1 μl) with 1 μl 4-HCCA matrix solution at 10 g/l in 50% MeCN/H2O/0.1% TFA. As MALDI-TOF linear mode measurement is well known barely inaccurate, myoglobin (Sigma) was added onto spotted BAF and pBAF as internal standard (using double and single charged Myoglobin mass species) for more accurate measurements. Ten measurements were performed from the same sample spot and mean masses with standard deviation were calculated.

Phosphorylation sites on pBAF were identified after V8-GluC enzymatic digestion of both proteins and after reflector mode MALDI-TOFTOF measurements, Peptide Mass Fingerprints (PMFs) and MSMS sequencing analyses. Therefore, BAF and pBAF samples were diluted at 20 μM in potassium phosphate buffer 50 mM pH 7.8 and submitted to proteolytic digestion with endo-GluC V8 ([E/S]:[1/10]:[w/w]) at 25°C for 24 h. Incubation was stopped by acidification with TFA 5% and digested samples were spotted onto MALDI plates with 4-HCCA matrix as above. PMFs of both samples were obtained on MALDI-TOF MS in reflector positive mode. m/z peaks of interest were selected for MSMS sequencing by PSD-MALDI-TOFTOF. All spectra were externally calibrated with commercial standard. MS/MS spectra annotations were performed manually.

#### X-ray crystallography

The pBAF dimer was purified by gel filtration on a Superdex 200 pg HiLoad 16/600 column in 50 mM Tris–HCl pH 8.0, 150 mM NaCl and concentrated to 20 mg/ml. The pBAF-EmN complex was purified by gel filtration on a Superdex 200 pg Increase 10/300 in 50 mM HEPES pH 7.4, 100 mM NaCl, 5 mM DTT and concentrated to 2 mg/ml. Crystallization experiments were carried out at the HTX Lab (EMBL Grenoble) (42). Crystals were flashed-freezed in liquid nitrogen and prepared for X-ray diffraction experiments using the CrystalDirect technology (43). pBAF crystals were obtained by sitting drop vapor diffusion at room temperature against reservoir containing 0.2 M sodium fluoride, 20% (w/v) PEG 3350. Crystals were soaked with 0.5 M cesium chloride diluted with the crystallization condition for about 5 min. Diffraction data were collected at the P14 beamline (Petra III synchrotron, Hamburg, Germany). The 3D structure was solved by molecular replacement with Molrep software in CCP4 using the 1CI4.pdb coordinates file as starting model (44,45). Crystals of pBAF-EmN complex were obtained by sitting drop vapor diffusion at room temperature against reservoir containing 0.1 M of HEPES pH 7.5 and PEG 6000 25% (w/v). Diffraction data were collected at the ID23 beamline (ESRF synchrotron, Grenoble, France). The 3D structure was solved by molecular replacement with PHASER from PHENIX suite using the 6GHD.pdb coordinates file as starting model (44,45). The resulting models were iteratively improved by alternating manual reconstruction with COOT software (46) and refinement with BUSTER software ((47); Supplementary Table S1). All structure representations were prepared using PyMOL (Schrodinger, LLC).

#### Protein-ligand interactions

In order to monitor BAF (WT, variants) binding to a 48-nt dsDNA coated on a grid, fluorescence experiments were performed on a DRX2 instrument (Dynamic Biosensors GmbH, Martinsried, Germany). The experiments were carried out on standard multipurpose chips (MPC2-48-2-G1R1-S) using the static measurement mode. The immobilized dye was excited in the range of 600–630 nm and emission in the range of 650–685 nm was recorded. Assays were carried out at 10°C in the TE140 buffer (10 mM Tris pH 7.4, 140 mM NaCl, 0.05% Tween-20, 50 μM EDTA, 50μM EGTA). Increasing amounts of BAF (from 1.6 to 200 nM), BAF A12T (from 3.9 nM to 1 μM), pBAF (from 5 to 160 μM), BAF S4E (from 1.6 to 100 μM), were added at a flow rate of 500 μl/min. Data analysis was performed using the Switch Analysis software provided with the instrument.

Isothermal Titration Calorimetry (ITC) experiments were performed using a high-precision VP-ITC calorimetry system at 15°C. To characterize interactions between the BAF dimers (WT, variants) and dsDNA (7-nt, 21-nt, 48-nt), the proteins were dialyzed against 50 mM Tris–HCl pH 8.0, 150 mM NaCl. BAF (WT, variants) at 20 μM in the cell was titrated with the 7-nt and 21-nt dsDNA at 200 μM or the 48-nt dsDNA at 100 μM in the injection syringe. To characterize interactions between BAF dimers (unphosphorylated or phosphorylated) and LamIgF or LEM fragments, all proteins were purified by gel filtration using the same buffer (50 mM HEPES pH 7.4, 150 mM NaCl). BAF or pBAF at 20 μM in the instrument cell was titrated with LamIgF at 100 μM or LEM at 150 μM in the injection syringe. Data analyses were performed using the Origin software provided with the instrument.

## RESULTS

### Phosphorylation by VRK1 induces a significant change in the NMR fingerprint of BAF in solution

**Figure 2.**
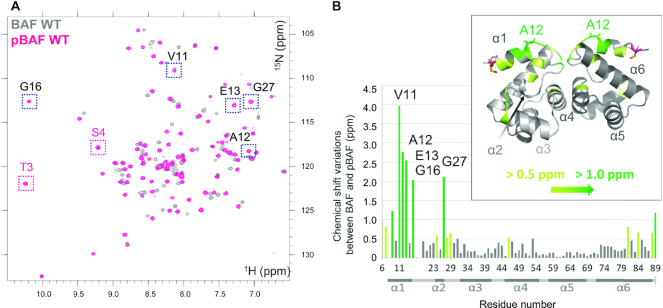
Phosphorylation by VRK1 significantly modifies the ^1^H–^15^N HSQC spectrum of BAF in solution. (**A**) Superimposition of the ^1^H–^15^N HSQC spectra of BAF before (grey) and after (pink) phosphorylation by VRK1. These spectra were obtained at pH7.2, 30°C and 700 MHz. Assignment of the ^1^H,^15^N NMR chemical shifts of pBAF revealed that the peaks corresponding to Thr3 and Ser4 (boxed in pink) are found in a spectral region typical for phosphorylated residues. (**B**) NMR chemical shift variations due to phosphorylation by VRK1. From the analysis of the NMR spectra displayed in (A), chemical shift variations due to phosphorylation were calculated for each residue, using the equation:$\;\sqrt {{{( {\delta H{N_{BAF}} - \delta H{N_{pBAF}}} )}^2} + {{( {\delta {N_{BAF}} - \delta {N_{pBAF}}} )}^2}/25}$. Bars corresponding to residues 2–5 are not shown because peaks corresponding to these residues were not observed on the BAF spectrum at pH 7.2 and 30°C. Chemical shift variations larger than 0.5 ppm and 1.0 ppm are indicated in yellow-green and green, respectively. A set of residues, including Ala12 mutated in NGPS (4,49), show variations larger than 2.0 ppm: they are boxed in black on the pBAF NMR spectrum in (A) and marked on the bar graph in (B). A 3D view of the chemical variations due to phosphorylation is inserted. In this picture, the 3D structure of non-phosphorylated BAF (PDB: 6GHD) was used to position the residues with large chemical shift variations due to phosphorylation by VRK1. Thr3, Ser4 and Ala12 are represented with sticks and Thr3 and Ser4 are in orange and pink, respectively. Other colors are as in the bar graph.

In order to identify the structural consequences of BAF phosphorylation by VRK1, we purified both a BAF construct in which the four cysteines are replaced by alanines, as reported in (29), and the catalytic domain of VRK1. Mixing the two proteins led to a clear shift of the SDS-PAGE band corresponding to BAF (Supplementary Figure S1A). Phosphorylation did not significantly impact the thermal stability of our BAF construct (Supplementary Figure S1B). We further recorded 2D NMR ^1^H–^15^N HSQC spectra of BAF and phosphorylated BAF (pBAF) at 30°C (Figure 2A). The number of peaks is similar in both spectra, demonstrating that pBAF corresponds to a unique phosphorylated species. However, the spectrum of pBAF (in pink) exhibits a large number of significant differences when compared to that of BAF (in dark grey).

The ^1^H–^15^N HSQC spectrum of BAF was already assigned by Clore and co-workers (40), but the important differences observed upon incubation with VRK1 forced us to produce a ^15^N, ^13^C labelled sample of pBAF, in order to record 3D NMR ^1^H, ^15^N, ^13^C HNCA, CBCACONH, HNCO and HNCACO experiments and assign its backbone NMR chemical shifts *de novo* (BMRB code: 50298). Half of the ^1^H–^15^N HSQC signals are shifting by more than 0.15 ppm upon phosphorylation (combined ^1^H and ^15^N shift value; see Figure 2B). These signals correspond to residues located in three regions of BAF: region from aa 6 to aa 33, forming helices α1 and α2, region from aa 43 to aa 53, forming helix α4 at the interface between BAF monomers, and region from aa 76 to aa 89, forming the C-terminal helix α6 (Figure 2B). We also noticed that the only serines and threonines resonating in the ^1^H–^15^N spectral region where phosphorylated serines and threonines are typically found are Thr3 and Ser4 (48). In the published crystal structures of BAF, Ser4 is the first residue of helix α1. Because NMR ^13^C chemical shifts variations induced by phosphorylation and helical structures are similar, we could not definitely confirm that these two residues were phosphorylated by VRK1 solely based on the analysis of their ^13^C_α_ and ^13^C_β_ chemical shifts. Hence, we used a complementary experimental approach to validate the identification of the phosphorylated residues.

### BAF is phosphorylated on Thr3 and Ser4

**Figure 3.**
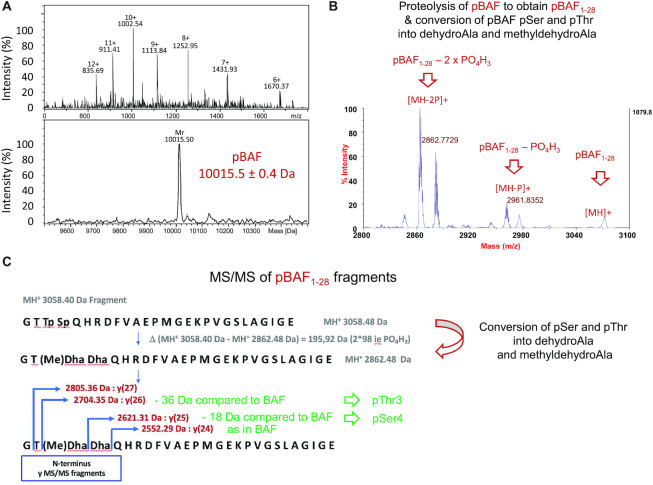
MS analyses confirm that pBAF is phosphorylated at Thr3 and Ser4. (**A**) Accurate mass measurement of pBAF obtained by μLC-ESI-MS. This analysis specified that the mass of pBAF is 10015.4 ± 0.6 Da. BAF did not give any multi-charged mass spectrum that could enable the measurement of its accurate molecular weight. (B, C) Identification of the phosphorylated residues by fragmentation and MS. Peptide Mass Fingerprint of pBAF digested by endo-GluC was obtained using MALDI-TOF MS in reflector positive mode. M/z peaks were then selected for MSMS sequencing by PSD-MALDI-TOFTOF. (**B**) Zoom view of the MSMS spectrum of the double phosphorylated BAF_1–28_ peptide at m/z 3058.40. Fragmentation of pBAF_1–28_ lead to the conversion of pSer and pThr into dehydroAla and methyldehydroAla, due to a β-elimination of the phospho-groups (56,57). (**C**) Further fragmentation of pBAF_1–28_ into *y*(*i*) peptides (containing ‘*i*’ amino acids) revealed that Thr3 and Ser4 are phosphorylated. The whole MS spectrum of fragmented pBAF_1–28_ is shown in Supplementary Figure S2B.

We performed mass spectrometry experiments (MS) on both BAF and pBAF. On-line μLC–ESI-MS experiments provided the mass of pBAF: 10015.5 ± 0.4 Da, which fitted with the theoretical mass of BAF with two phosphorylated residues (10 016 Da; Figure 3A). MALDI-TOF experiments confirmed that the mass difference between BAF and pBAF (162 ± 2 Da) corresponds to two phosphorylation events (Supplementary Figure S2). In order to identify the phosphorylated residues, Peptide Mass Fingerprint was performed on both proteins after endoproteinase GluC digestion. This analysis yielded a candidate phosphorylated fragment assigned to the N-terminal peptide of pBAF from aa 1 to aa 28 (*m*/*z* 3058.5 Da, i.e. with a mass increment of 159.9 Da compared to the unphosphorylated peptide, data not shown). MALDI-TOFTOF MS/MS sequencing of this fragment and manual analysis of the resulting daughter-ions spectrum unambiguously revealed that pBAF is phosphorylated on Thr3 and Ser4 (Figure 3B and C). Thus, resonances of Thr3 and Ser4 observed after incubation of BAF with VRK1 do correspond to phosphorylated Thr3 (pThr3) and Ser4 (pSer4), which is consistent with our NMR results.

### VRK1 first phosphorylates Ser4 and then Thr3

**Figure 4.**
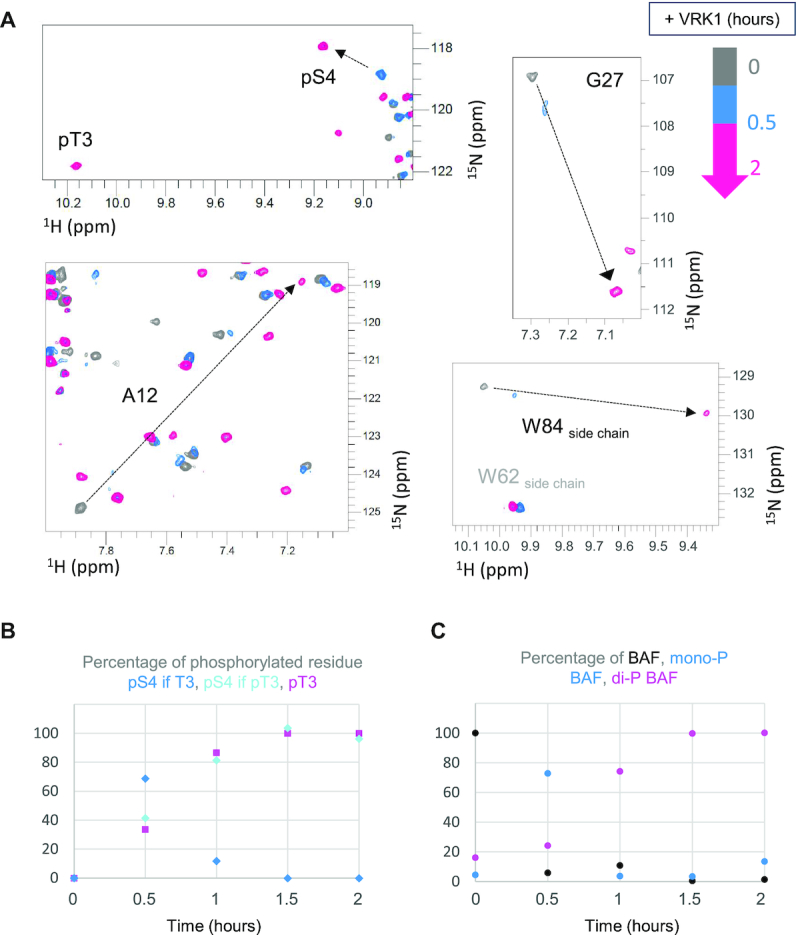
Real-time NMR monitoring of BAF phosphorylation. 2D NMR ^1^H–^15^N HSQC spectra were recorded every 30 min after addition of VRK1 at pH7.2, 30°C and 700 MHz. (**A**) Four selected zoom views from the superimposition of the spectra acquired at 0 (grey), 0.5 (blue) and 2 (pink) hours. The black arrows identify peaks largely shifted due to phosphorylation. (**B**) Percentages of phosphorylation as a function of time deduced from the intensities of the peaks corresponding to pThr3 and pSer4. Whereas this percentage is close to 100% at the end of the kinetics for pThr3 and pSer4 in the final di-phosphorylated BAF species (cyan and pink curves), it increases up to 75% and then decreases down to 0% in the case of pSer4 in the intermediate, mono-phosphorylated BAF species. (**C**) Percentages of BAF, mono-phosphorylated BAF and di-phosphorylated BAF, as a function of time. Here, the phosphorylation kinetics was monitored from the intensities of the peaks corresponding to selected residues, i.e. Ala12, Gly27 and the side-chain of Trp84. These peaks are largely shifted upon phosphorylation, as shown in (A). Three positions can thus be observed for each of these peaks, corresponding to BAF, monophosphorylated BAF and di-phosphorylated BAF. The peak intensities were followed at each of these positions with time, translated into percentages and averaged upon the three residues.

Having identified the phosphorylated residues, we searched for the phosphorylation event responsible for the large changes observed in the pBAF 2D NMR ^1^H–^15^N HSQC spectrum. Therefore, we monitored the NMR backbone ^1^H, ^15^N signals of BAF over time in a time series of ^1^H–^15^N HSQC spectra recorded during the phosphorylation reaction (Figure 4A). We distinguished three sets of peaks in total, which we assigned to non-phosphorylated BAF, mono-phosphorylated BAF and di-phosphorylated BAF (pBAF). In a first step, the initial set of ^1^H–^15^N cross-peaks (in dark grey) disappeared, whereas a second set of peaks appeared, after 30 min (in blue) in our conditions. No large shift was observed for most peaks between the grey and blue spectra (Supplementary Figure S3A), except in the case of a few peaks exemplified in the zoom views (Figure 4A). A third set of peaks, corresponding to pBAF, was observed after 60 min (in pink). As noticed above, the spectrum of pBAF exhibited important differences compared to the spectra of BAF and mono-phosphorylated BAF. These differences were particularly large again for the peaks shown in the zoom views (Figure 4A).

Furthermore, we noticed that the spectrum of the mono-phosphorylated BAF exhibited a strong peak in the vicinity of the pBAF peak corresponding to pSer4, and did not reveal any peak in the vicinity of the pBAF peak corresponding to pThr3. This suggested that the mono-phosphorylated BAF was phosphorylated on Ser4 (Figure 4A, upper left zoom view). We measured the intensities of the peaks assigned to pSer4 when Thr3 is not phosphorylated, pSer4 when Thr3 is phosphorylated and pThr3 and plotted them as a function of time (Figure 4B). Consistently with our analysis, the peak corresponding to pSer4 in mono-phosphorylated BAF was intense at 0.5 h and then decreased with time, whereas the peaks corresponding to pSer4 and pThr3 in pBAF increased in order to reach 100% at the end of the kinetics. The peaks corresponding to the N-terminal residues from aa 1 to aa 5 of BAF were not observed at pH 7.2 and 30°C, precluding analysis of the decays of the peaks corresponding to Thr3 and Ser4 over time. Thus, we selected three other residues (identified in the zoom views of Figure 4A) whose peaks significantly shifted upon phosphorylation. We measured their intensities on each spectrum, and from these values, the percentages of BAF, mono-phosphorylated BAF and pBAF were plotted as a function of time (Figure 4C). This analysis confirmed that most BAF molecules were already mono-phosphorylated after 0.5 h, and that they were di-phosphorylated after 1.5 h.

We monitored by NMR the phosphorylation of the BAF variants T3A and S4E (Supplementary Figure S3B). Whereas the spectrum of phosphorylated BAF T3A is similar to that of mono-phosphorylated BAF (left view), the spectrum of phosphorylated BAF S4E is similar to that of pBAF. However, the chemical shift variations due to phosphorylation are systematically smaller in the case of BAF S4E compared to BAF WT (right view). Altogether, we concluded that VRK1 phosphorylated first Ser4, then Thr3, and that phosphorylation of Thr3 triggers large changes in the ^1^H–^15^N HSQC spectrum of BAF, which are increased when Ser4 is also phosphorylated.

### Di-phosphorylated BAF adopts a 3D structure similar to that of BAF, but is significantly less flexible in solution

**Figure 5.**
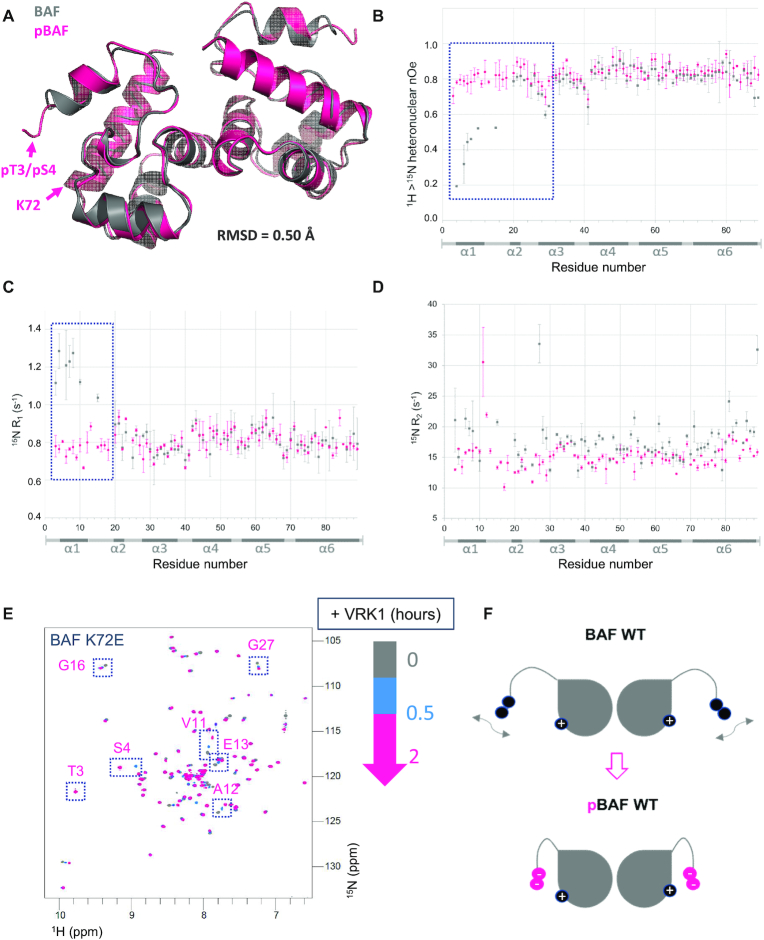
BAF and pBAF share similar 3D structures but exhibit different dynamics in solution. (**A**) X-ray structure of BAF phosphorylated by VRK1, superimposed onto the structure of non-phosphorylated BAF. Both structures are displayed as cartoons, pBAF being colored in pink and BAF (6GHD) in grey. The positions of pThr3, pSer4 and Lys72 in pBAF are indicated; their side chains cannot be unambiguously positioned in the electron density of pBAF due to lack of resolution. The specified RMSD value was calculated between all atoms of BAF and pBAF. The statistics of the pBAF structure are presented in Supplementary Table S1. (**B**–**D**) ^15^N relaxation data recorded on BAF and pBAF as a function of the residue number. ^15^N R_1_, R_2_ relaxation rates and ^1^H→^15^N nOe of BAF and pBAF, recorded at pH 7.0, 293K and 700 MHz, are displayed in grey and pink, respectively. Regions in which large differences are observed are highlighted by dotted boxes. (**E**) NMR monitoring of the phosphorylation kinetics of the BAF variant K72E. The ^1^H–^15^N HSQC spectra recorded at 0, 0.5 and 2 h after addition of VRK1 are displayed in grey, blue and pink, respectively (as in Figure 4A). Peaks that largely shifted due to phosphorylation in BAF WT (see Figure 2A) are boxed. (**F**) Model highlighting how the flexibility of region from amino acid 1 to amino acid 18 in BAF (before Pro19) is reduced in pBAF due to electrostatic interactions between the phosphorylated residues and residues from helix α6 including Lys72.

In order to obtain a detailed view of the structural consequences of BAF phosphorylation, we crystallized pBAF and solved its 3D structure at a resolution of 3.2 Å (Supplementary Table S1). While NMR revealed large chemical shift changes between BAF and pBAF, we did not observe any major structural changes due to phosphorylation in the crystal (Figure 5A). Only helix 1 is significantly shortened in pBAF compared to BAF: it comprises residues 4–12 in BAF and is restricted to residues 7–11 in pBAF. And only residues Thr3, Ser4 and Gln5 have a Cα atom displaced by more than 2 Å after fitting the two structures.

However, comparison of the dynamics of BAF and pBAF in solution by NMR revealed that the motions observed in BAF are strongly reduced in pBAF (Figure 5B–D). First, the N-terminal region from Thr3 to Phe10 (comprising helix α1), as well as Met15, shows significantly increased ^15^N R_1_ but decreased ^1^H→^15^N nOe values in BAF compared to pBAF. This indicated that, in BAF, the N-terminal region is flexible on a picosecond to nanosecond timescale, whereas such N-terminal motions are not present in pBAF. In general, the ^1^H→^15^N nOe values measured in pBAF are mostly close to 0.8, which demonstrates that pBAF is rigid on a fast timescale. Concerning ^15^N R_2_ values, in BAF, helix α1 exhibits high relaxation rates, and residues Val11 to Glu13 and Gly16 to Lys18, did not even yield detectable amide H-N cross-peaks, which precluded measurements of their ^15^N relaxation properties. This shows that helix α1 and loop α1α2 exhibit slow motions on a microsecond to millisecond time scale. In pBAF, only Val11 and Ala12 exhibit large ^15^N R_2_ values; as these residues are packed against Phe88, the mobility of the Phe88 ring could be responsible for the large relaxation rates. Helix α4 at the interface between the two monomers and helix α6 at the C-terminus also have increased R_2_ values compared to pBAF, whereas their R_1_ values and nOe values are similar: these helices exhibit slow (micro- to millisecond time scale) motions in BAF that are not observed in pBAF. In summary, BAF is particularly flexible in helix α1 and loop α1α2 (residues 3 to 18), and exhibits additional slow motions in helix α4, at the interface between the 2 monomers, and in helix α6, interacting with the N-terminal region, whereas pBAF mainly senses slow motions in the vicinity of loop α1α2.

Thus, phosphorylation of Ser4 and Thr3 drastically reduces the conformational mobility of BAF. As pThr3 and pSer4 are close to positively charged residues in helix α6 (Lys72, Arg75), electrostatic interactions between the phosphorylated side chains and helix α6 could explain the strong decrease in pBAF flexibility (Figure 5A). We hypothesized that a subpopulation of conformations of BAF in which the N-terminal region of BAF is close to helix α6 is enriched after phosphorylation, which explains the spectral changes triggered by phosphorylation of Thr3 (in the presence of S4E or pSer4). In order to test this hypothesis, we monitored phosphorylation of the BAF mutant K72E by NMR (Figure 5E). We observed that the large spectral change triggered by phosphorylation of Thr3 in BAF WT does not occur in BAF K72E. Thus, electrostatic interactions between the phosphorylated side chains and positively charged residues of helix α6, including Lys72, contribute to the conformational selection triggered by phosphorylation (Figure 5F).

### Phosphorylation of BAF strongly reduces binding to DNA, but does not affect binding to lamin A/C and emerin

**Figure 6.**
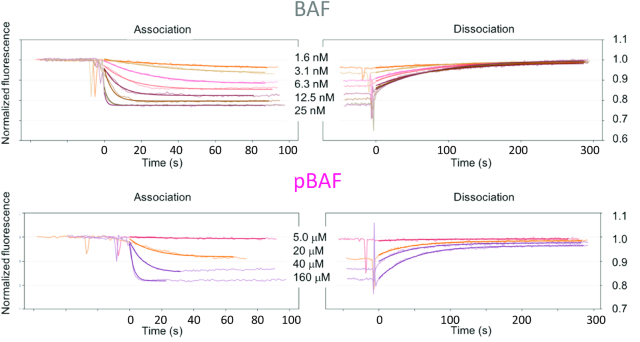
Phosphorylation of BAF by VRK1 greatly reduces its affinity for dsDNA. Fluorescence experiments revealed that the affinity of BAF WT for a coated 48 nt dsDNA is 2.5 ± 1 nM, whereas the affinity of pBAF for this same dsDNA is 11 ± 2 μM. Binding curves of BAF variants S4E and A12T are displayed in Supplementary Figure S4 and all affinities are summarized in Table 1.

We aimed at describing the functional impact of BAF phosphorylation and associated structural perturbations. BAF directly interacts with DNA, lamin A/C and emerin. The binding interfaces of BAF with DNA and lamin A/C includes its N-terminal region (Figure 1B), which exhibits large chemical shift variations upon phosphorylation (Figure 2B), associated with variations in dynamics in solution (Figure 5). At the opposite, its interface with emerin is distant from its phosphorylation sites and exhibits no chemical shift variation upon phosphorylation (Figure 2B). Thus, we hypothesized that phosphorylation affects BAF binding to DNA and lamin A/C. It was already reported that pBAF is defective for binding to DNA (15,25,33). To quantify this, we measured the affinities of BAF and pBAF for double-stranded DNA (dsDNA) by monitoring the fluorescence of a dye attached to a 48-nucleotides (nt) dsDNA coated on a grid. The apparent affinities of BAF and pBAF for dsDNA are 2.5 ± 1 nM and 11 ± 2 μM, respectively (Figure 6; Table 1). Moreover, whereas the NGPS-associated BAF A12T has the same affinity for dsDNA as BAF WT, the phosphomimetic BAF S4E has the same affinity as pBAF (Supplementary Figure S4; Table 1). To confirm these results in solution, we performed Isothermal Titration Calorimetry (ITC) experiments: the apparent *K*_d_ of BAF and BAF A12T for dsDNA are similar and depend on the length of the dsDNA, ranging from ∼1 μM for a 7nt-, to ∼0. 1 μM for a 21nt- and ∼35 nM for a 48nt- dsDNA (Table 1). In contrast, BAF S4E shows no detectable affinity for 7nt- and 21nt- dsDNA using this technique. Thus, regarding DNA binding, BAF S4E is an appropriate phosphomimic, suggesting that phosphorylation of Ser4 is the event responsible for the loss of affinity of BAF for dsDNA.

**Table 1.** Summary of the binding affinities measured between BAF WT and variants as well as pBAF and different fragments of dsDNA. The fluorescence experiments were performed between 2 and 5 times and provided affinities for a coated 48 nt dsDNA. ITC experiments were performed in solution using dsDNA fragments of 7 nt, 21 nt and 48 nt (n.b.: no detected binding; -: no data). They were all recorded in duplicate. All the affinities are in M^-1^.

	**FLUORESCENCE EXPERIMENTS with a 48 nt dsDNA**						
	1-Kd	2-Kd	3-Kd	4-Kd	5-Kd	**Average Kd**	Standard Dev
**BAF WT**	2.10E-09	1.40E-09	4.20E-09	2.20E-09	2.80E-09	**2.54E-09**	1.05E-09
**BAF A12T**	1.60E-09	1.70E-09	3.70E-09	3.00E09	-	**2.50E-09**	1.02E-09
**BAF S4E**	17.0E-06	13.0E-06	-	-	-	**15.0E-06**	2.83E-06
**pBAF**	12.0E-06	9.40E-06	-	-	-	**10.7E-06**	1.84E-06
	**ITC EXPERIMENTS with a 48 nt dsDNA (ave. stoich. 0.2)**			**ITC EXPERIMENTS with a 21 nt dsDNA (ave. stoich. 0.3)**		**ITC EXPERIMENTS with a 7 nt dsDNA (ave. stoich. 0.9)**	
	1-Kd	2-Kd	**Average Kd**	1-Kd	2-Kd	1-Kd	2-Kd
**BAF WT**	37.7E-09	44.4E-09	**41.1E-09**	0.17E-06	0.11E-06	1.00E-06	2.10E-06
**BAF A12T**	37.0E-09	20.6E-09	**28.8E-09**	0.30E-06	0.21E-06	1.70E-06	0.92E-06
**BAF S4E**	-	-	-	n.b.	n.b.	n.b.	n.b.
**pBAF**	-	-	-	-	-	-	-

**Figure 7.**
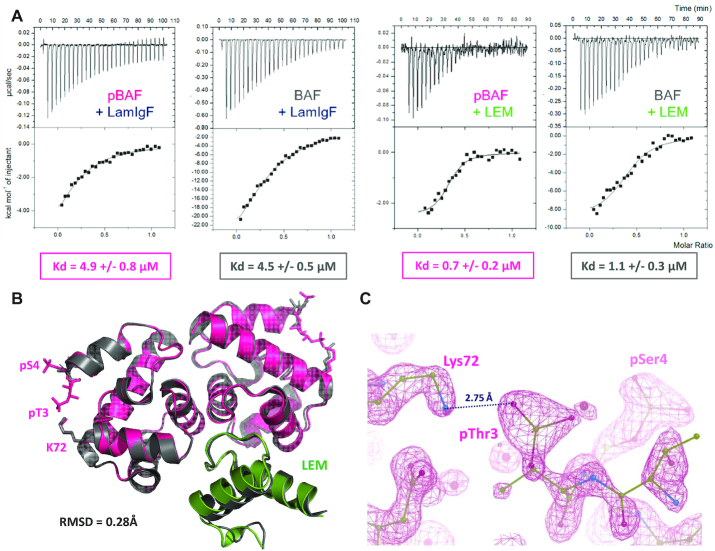
pBAF still forms a ternary complex with both lamin A/C and emerin. (**A**) ITC analysis of the interactions between pBAF and the lamin LamIgF or the emerin LEM fragments. pBAF (or BAF for comparison) being in the cell, the fragment LamIgF or LEM was injected and the heat signal was measured as a function of the injection number. Experiments were performed in duplicate, as detailed in Supplementary Table S2. (**B**) X-ray structure of pBAF bound to the LEM domain of emerin. The 3D structure of the complex is displayed as a cartoon, pBAF being in pink and the LEM domain in green. It is superimposed with that of BAF bound to the LEM domain (in grey; PDB 6GHD; (29)). The side chains of residues Thr3, Ser4 and Lys72 are displayed in sticks in both complexes. The specified RMSD value was calculated between all atoms of the pBAF-LEM and BAF-LEM complexes. The statistics of the pBAF-LEM structure are presented in Supplementary Table S1. (C) Zoom view on the electron density map defining the position of the side chains of pThr3, pSer4 and Lys72 in pBAF.

In order to further test binding of pBAF to lamin A/C, we measured the affinity of this interaction by ITC. We found that BAF and pBAF bind to the lamin fragment LamIgF, including the Igfold domain, with a *K*_d_ of 4.5 ± 0.5 and 4.9 ± 0.8 μM, respectively (Figure 7A; Supplementary Table S2). The binding enthalpy is significantly larger in the case of BAF compared to pBAF. However, it is compensated by a strongly unfavorable entropic contribution. Thus, we demonstrated that phosphorylation of BAF does not interfere with lamin A/C binding, despite its impact on the flexibility of the N-terminal region interacting with lamin A/C.

We also verified that pBAF still interacts with the LEM domain of emerin. We observed by ITC that both BAF and pBAF bind with a K_d_ of about 1 μM to the purified LEM domain (Figure 7A). Here again, the binding enthalpy is significantly larger in the case of BAF compared to pBAF, but the entropic contribution is weak in the case of BAF whereas it is large and favorable in the case of pBAF (Supplementary Table S2). We even obtained crystals of the complex between pBAF and EmN that diffracted at a resolution of 1.4 Å (Supplementary Table S1). Determination of the high-resolution structure of the complex confirmed that BAF and pBAF adopt similar 3D structures in the crystal (Figure 7B). At this resolution, the two phosphorylated side-chains as well as the Lys72 side chain were unambiguously positioned, and a salt-bridge between the side chains of pThr3 and Lys72 was observed (Figure 7C). On the emerin side, only the LEM domain is visible in the crystal, the disordered region of EmN being probably proteolyzed during crystallogenesis, as previously reported (29). The position of the LEM domain on pBAF is similar to that observed for the LEM domain on BAF, as illustrated in Figure 7B.

Finally, we confirmed that pBAF is able to simultaneously bind to lamin A/C and emerin. Therefore, we recorded 2D NMR ^1^H–^15^N HSQC spectra on the ^15^N labelled fragment LamIgF alone, in the presence of pBAF, and in the presence of pBAF and emerin LEM or EmN (Supplementary Figure S5). Analysis of these experiments showed that the LamIgF fragment binds to pBAF through a β-sheet surface already identified as interacting with BAF (29). Further loss of intensity of all ^1^H–^15^N HSQC peaks corresponding to the globular Igfold domain upon addition of LEM or EmN confirmed that the pBAF-Igfold complex binds to the nucleoplasmic region of emerin. As controls, we verified that LamIgF binds to BAF S4E and to the complex between BAF S4E and EmN, but not to EmN alone (Supplementary Figure S5).

## DISCUSSION

BAF is an essential DNA-binding protein, important for genome integrity and mutated in a progeria syndrome (4,49). In dividing metazoan cells, the regulated formation of complexes between BAF, DNA, LEM-domain proteins and lamins controls nuclear breakdown, mitotic spindle assembly and positioning, as well as nuclear envelope reformation (7,8,14,15,18,50). The vaccinia-related kinases, belonging to the casein kinase family, phosphorylate BAF at Ser4, which is required for nuclear envelope disassembly and facilitates BAF release from chromatin (19,33,35). *In vitro*, mitotic phosphorylation of human BAF by VRK1 reduces its interaction with DNA (15,25,33). Several teams also suggested that it reduces BAF binding to LEM-domain proteins and lamin A/C, thus contributing to disrupt anchorage of chromosomes to the nuclear envelope in mitosis as well as in meiosis (33,34,36,51). A recent study performed in *Drosophila* muscle fibers proposed a different analysis, in which BAF localization at the nuclear envelope requires the activity of VRK1, and non-phosphorylatable BAF is excluded from the nuclear envelope (52). We previously reported the 3D structure of BAF bound to the emerin LEM domain and the lamin A/C Igfold domain, highlighting the position of Ser4 close to the interface with lamin A/C (Figure 1B; (29)). We here aimed at explaining the impact of phosphorylation by VRK1 on BAF structural and binding properties at the molecular level.

### BAF phosphorylated by VRK1 exists as mono- and di-phosphorylated species

We first described VRK1-dependent phosphorylation kinetics of BAF at the residue level. Bengtsson & Wilson had already revealed that, if BAF is mostly unmodified in unsynchronized HeLa cells, two other phosphorylated or acetylated species can still be detected, and that BAF is hyperphosphorylated in mitosis (34). Moreover, it has been very recently demonstrated that in *Drosophila* cells, BAF is both mono- and di-phosphorylated, and that overexpression of VRK1 increases the proportion of di-phosphorylated BAF (14). Finally, using human recombinant BAF and several variants mutated at Thr2, Thr3 and Ser4, it was shown *in vitro* that Ser4 is the preferred VRK1 phosphorylation site, and Thr2 and/or Thr3 are also phosphorylated by VRK1, with a rate that is further slowed down due to phosphorylation of Ser4 (33). Using real-time NMR monitoring, we consistently observed that, *in vitro*, BAF is phosphorylated by VRK1 on Ser4, and then on Thr3. Residues Thr3 and Ser4 are strictly conserved in BAF, whereas Thr2 is not (Figure 1A). We did not observe any phosphorylation of Thr2 in BAF, neither by NMR or mass spectrometry. However, we did observe a small population of BAF T3A phosphorylated on Thr2 (Supplementary Figure S3B), showing that a mutation close to the phosphorylation sites can modify the kinase target. Combining all the results available from our work and the literature on WT BAF, we propose that phosphorylation by VRK1 creates both mono- (pSer4) and di-phosphorylated (pSer4 and pThr3) species that might co-exist in cells.

### The high flexibility of BAF is strongly reduced after phosphorylation on both Ser4 and Thr3

We then characterized the structural differences between non-phosphorylated, mono-phosphorylated and di-phosphorylated BAF. We showed that the crystal structures of BAF and di-phosphorylated (pBAF) are similar. However, in solution, BAF exhibits large motions on both fast (picosecond to nanosecond) and slow (microsecond to milliesecond) timescales, which are not observed in pBAF. In particular, the N-terminal helix α1 and loop α1α2 of BAF are flexible on a large timescale range, as revealed by their high ^15^N R_1_ and R_2_ and low ^1^H→^15^N nOe values. Human BAF has 4 cysteines in helices α5 and α6, which are buried but still highly sensitive to oxidation, as reflected by the specific BAF purification protocols developed by several teams (1,15,33,34,40,53). In our construct, the cysteines are mutated into alanine, which reduces the thermal stability of the protein (40), but (i) three of the four cysteines are replaced by alanine in a set of organisms, in particular nematodes (Figure 1A), (ii) we verified that in all our conditions, the melting temperature of our BAF construct was higher than 40°C (see for ex Supplementary Figure S1B). The sensitivity of BAF buried cysteines to oxidation might be partially due to the large flexibility that we observed in several regions of BAF, particularly in its N-terminal region that is packed against helix α6. We here demonstrated that BAF di-phosphorylation strongly reduces BAF flexibility. Comparison of the NMR ^1^H–^15^N HSQC of BAF, the phosphomimetic BAF S4E, the phosphorylated BAF S4E and pBAF shows that increasing the negative charge of BAF N-terminal region progressively shifts BAF conformations towards a more rigid and unique structure, as observed for pBAF (Supplementary Figure S3A and B). In BAF T3A and K72E, the phospho-dependent shift is strongly reduced: when phosphorylated, both mutants have an NMR fingerprint that still corresponds to a flexible BAF structure (Figure 5E and Supplementary Figure S3B). Consistently, a salt-bridge is present between pThr3 and Lys72 side chains in the 3D structure of pBAF bound to the LEM domain of emerin (Figure 7C). Therefore, we conclude that the second phosphorylation event, as well as the presence of positively charged residues in the C-terminal helix α6, are essential to restrict the motions in BAF, and especially to restrict the position of the N-terminal region, including helix α1 and loop α1α2, relatively to the core helices α4 to α6 (Figure 5F).

### What are the functions of the two identified phosphorylation events on Ser4 and then Thr3?

The phosphomimetic BAF S4E, cited in several studies, only mimics the first phosphorylation event. However, we showed here that it is sufficient to reproduce the severe affinity decrease measured between pBAF and DNA (about 5000-fold when compared to BAF). The functional role of the second phosphorylation event, associated to the conformational shift, is less clear. Several studies suggested that phosphorylation by VRK1 decreases BAF binding to nuclear envelope proteins (33,34). In particular, blot assays performed on nitrocellulose membranes detected a weaker interaction between recombinant prelamin A tails and BAF after mutating Ser4 into Glu or incubating BAF in mitotic extracts, together with a weaker interaction between prelamin A tails (aa 394 to aa 664) and emerin in the presence of BAF S4E compared to BAF WT (34). It was also reported that immobilized emerin from aa 1 to aa 222 (a larger construct than EmN, which is not soluble at the concentrations that we are using) binds weaker to BAF S4E than to BAF WT (34), and a pull-down assay revealed that the emerin LEM domain shows a modest loss of affinity for pBAF when compared to BAF (33). Here, we could not detect any significant affinity decrease due to BAF phosphorylation, when tested against LamIgF (lamin A/C aa 411 to aa 566) and LEM (emerin aa 1 to aa 46) in solution by ITC (Figure 7A). We observed significant decreases in the (favorable) enthalpic contributions to the LamIgF and LEM binding energies due to phosphorylation; however, these decreases are compensated by weak and large favorable entropic contributions in the case of LamIgF and LEM, respectively. The increased dynamics of BAF versus pBAF in solution might explain the large differences in the binding thermodynamics observed between BAF and pBAF in this study. Nevertheless, these differences do not lead to any detectable affinity variation. From a structural point of view, NMR and X-ray crystallography analyses did not reveal any significant variation due to phosphorylation in the LEM binding region, which is far from Thr3 and Ser4 (Figures 2B and 5A). Determination of the high-resolution 3D structure of the complex between pBAF and the emerin LEM domain confirmed that BAF and pBAF bind similarly to this domain (Figure 7B). Furthermore, NMR titration experiments showed that pBAF as well as BAF S4E are still able to form a ternary complex together with LamIgF and EmN (or the LEM domain) (Supplementary Figure S5). Thus, using purified proteins and biophysical tools as ITC, NMR and X-ray crystallography, we could not identify a phospho-dependent decrease in the affinity of BAF for these nuclear envelope protein fragments. Additional contacts between BAF, lamin A/C and emerin, either mediated by protein regions different from LamIgF and EmN (54), or mediated by other biomolecules as for example DNA (55), could be weakened due to BAF phosphorylation in cells.

In conclusion, we performed a molecular analysis of BAF phosphorylation by VRK1 that is necessary for proper mitotic progression, as summarized in a short movie (https://youtu.be/Mn2o20m-J3g). We revealed that the N-terminal region of BAF is flexible in solution, which facilitates phosphorylation and dephosphorylation of Thr3 and Ser4. Phosphorylation of these residues by VRK1 does not induce large conformational changes in BAF. Our analysis suggests that phosphorylation of the conserved Ser4 is sufficient to strongly decrease the affinity of BAF for dsDNA. However, a second conserved residue, Thr3, is subsequently phosphorylated by VRK1 *in vitro*, which was also observed in cells. Our results indicate that this second event strongly shifts the conformation of BAF towards a more rigid structure, due to electrostatic interactions between the N-terminal phosphorylated helix α1 and the C-terminal positively charged helix α6. Unexpectedly, the di-phosphorylated BAF is still able to bind to its two well-characterized partners at the nuclear envelope: lamin A/C and emerin. Our study raises the question of the functional role of this second phosphorylation event, associated to a change in the distribution of BAF conformations in solution. Locking BAF conformation could disfavor dephosphorylation by PP2A-B56 (36) and/or prevent binding to yet unidentified partners. It could also regulate the formation of large oligomeric BAF species, which were observed *in vitro* by us and others (6), and whose function is still unknown.

## DATA AVAILABILITY

The NMR backbone chemical shifts of phosphorylated BAF were deposited at the Biological Magnetic Resonance Data Bank (BMRB code 50298; http://www.bmrb.wisc.edu/data_library/summary/index.php?bmrbId=50298). The crystal structures of phosphorylated BAF alone and bound to EmN were deposited at the Protein Data Bank, under the references 7ABM and 7DNY.

## Supplementary Material

gkab184_Supplemental_Files
