# Supplementary material for: Di-phosphorylated BAF shows altered structural dynamics and binding to DNA, but interacts with its nuclear envelope partners
Source: Nucleic Acids Res. 2021 Mar 21;49(7):3841–55. doi: 10.1093/nar/gkab184 (PMC8053085; doi:10.1093/nar/gkab184)
Supplement: gkab184_Supplemental_Files [file gkab184_supplemental_files.zip › figures-supp-review-NAR-3feb.pdf]

Suppl. figure 1

A

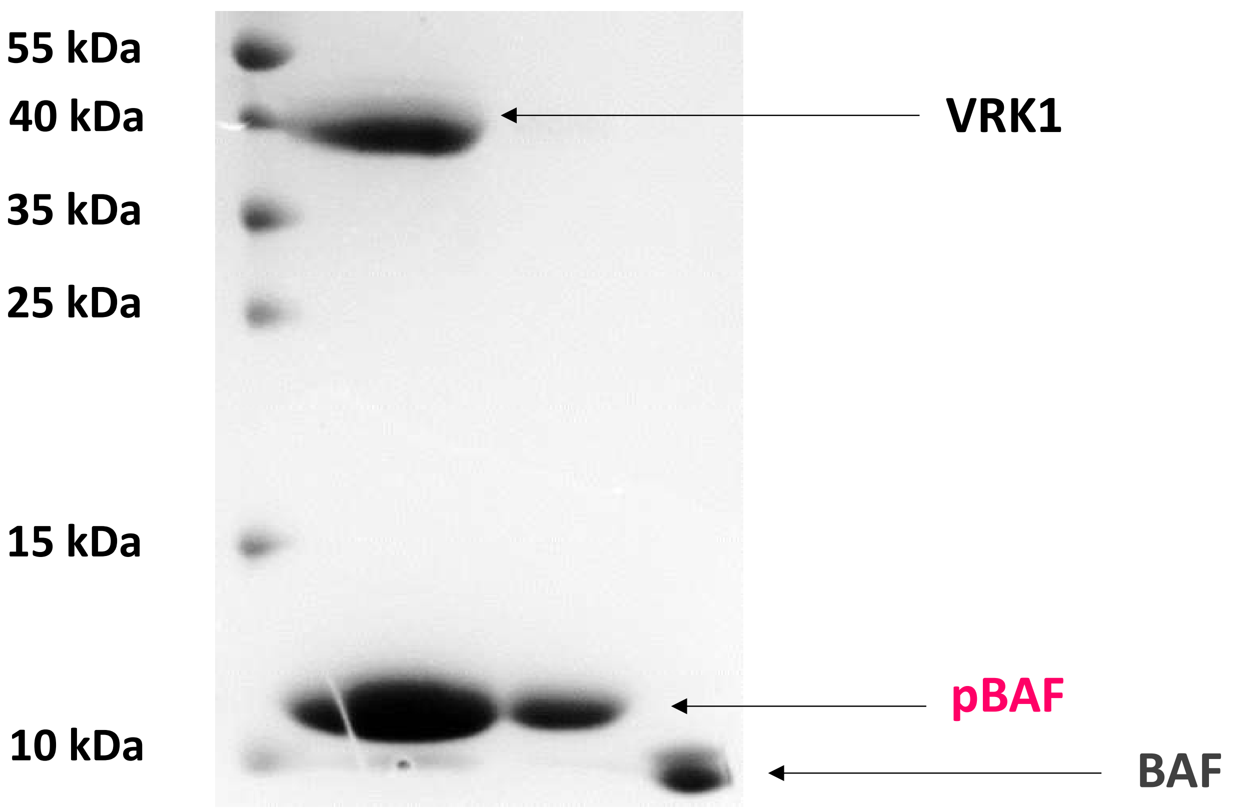

B

pBAF 50 mM Tris–HCl pH 8.0, 150 mM NaCl: 47°C

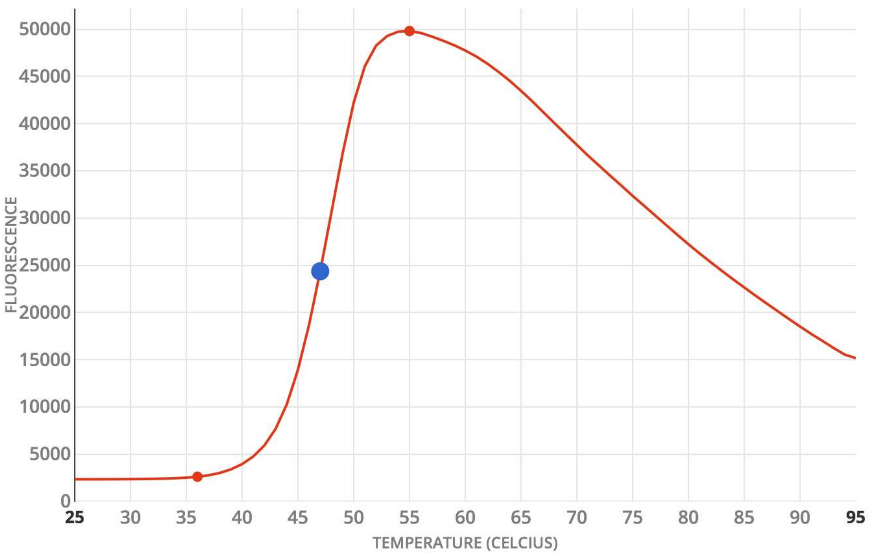

For comparison:
BAF 50 mM Tris–HCl pH 8.5, 100 & 200 mM NaCl: 44° & 45.5°C
50 mM NaP pH 7.5, 100 & 200 mM NaCl: 45.5° & 46.5°C

Suppl. figure 2

A

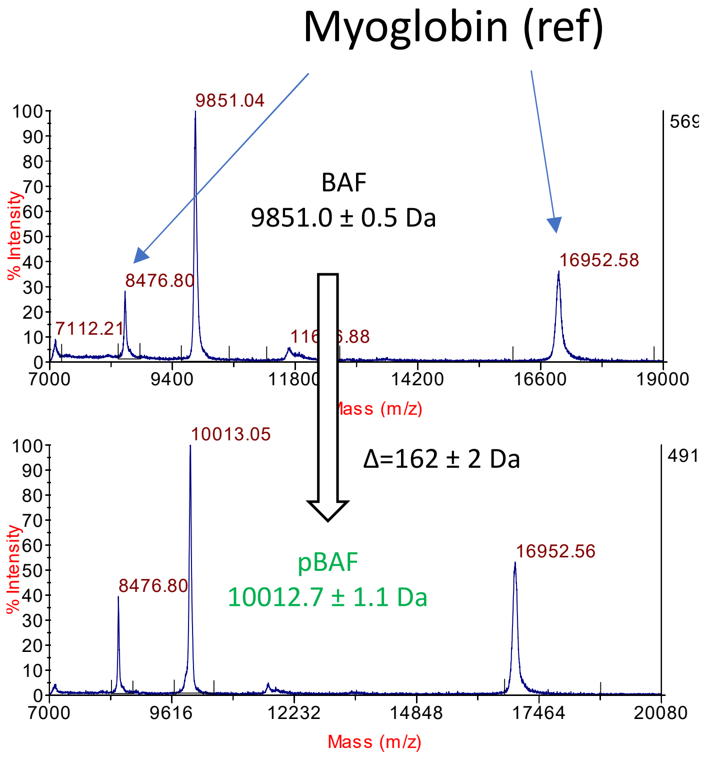

B

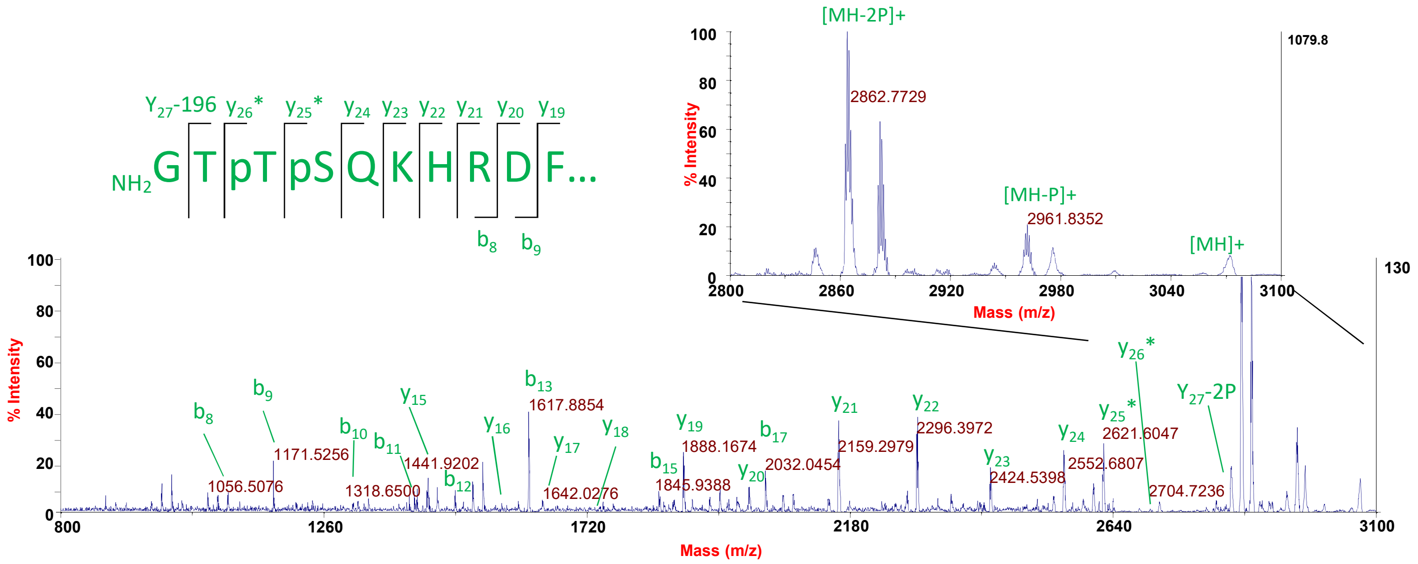

Suppl. figure 3

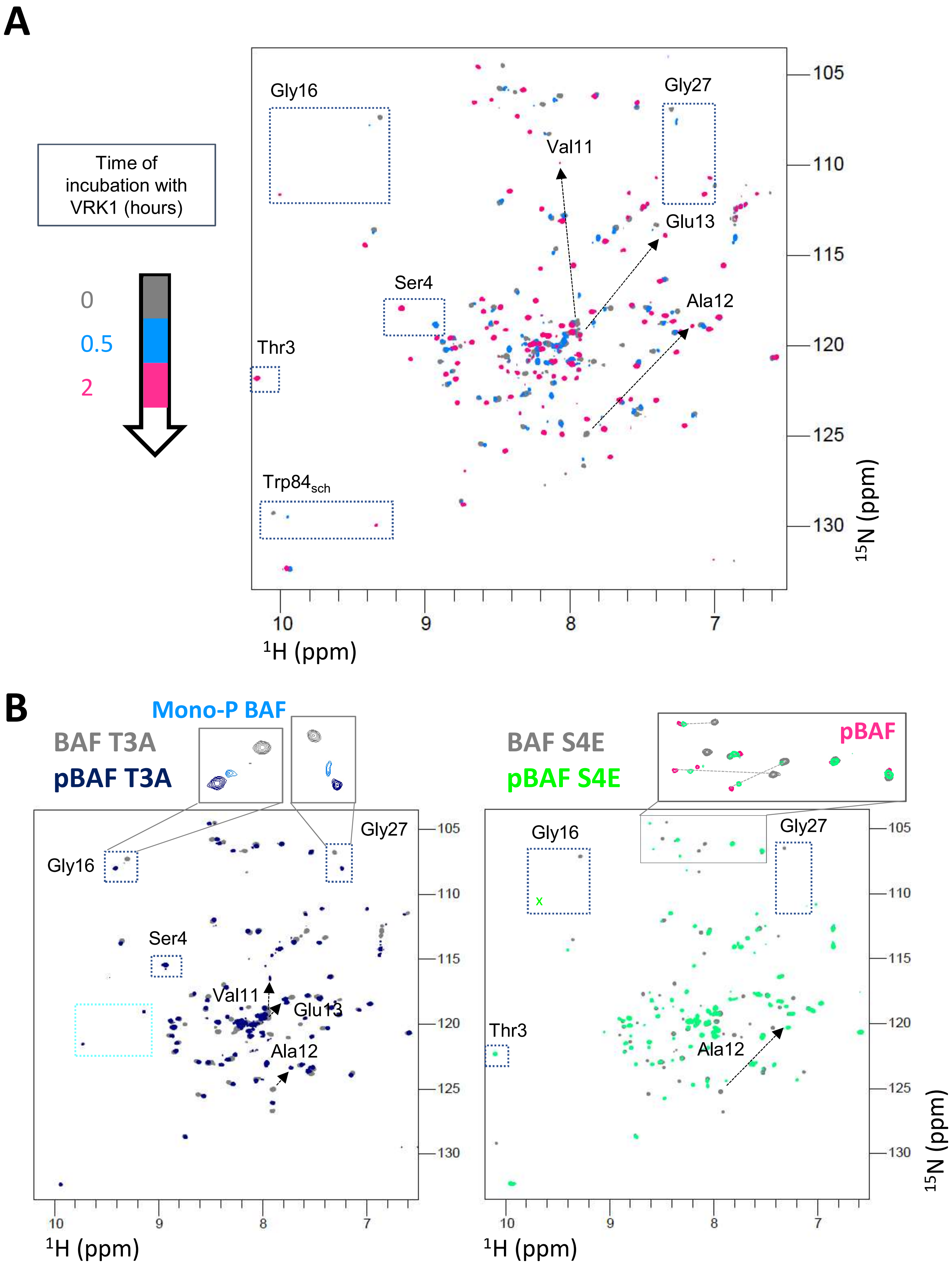

Suppl. figure 4

BAF WT

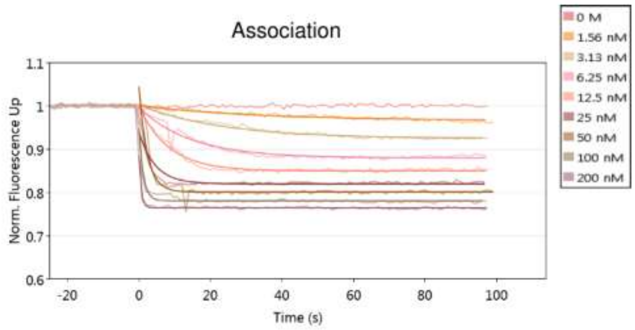

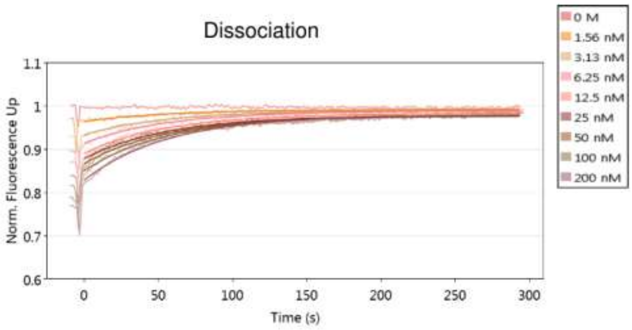

k_{ON} = 8.23 ± 0.20 E+6 $M^{-1} \cdot s^{-1}$

K_D = 2.16 ± 0.05 nM

k_{OFF} = 1.77 ± 0.01 E-2 s^{-1}

BAF A12T

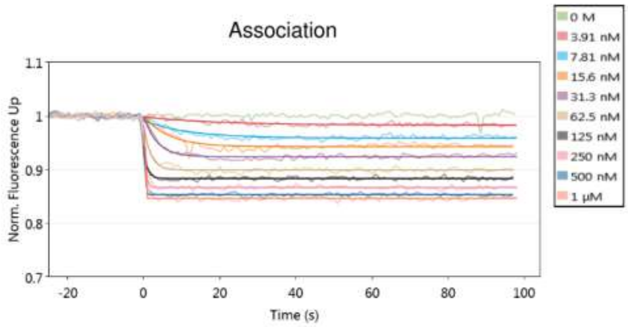

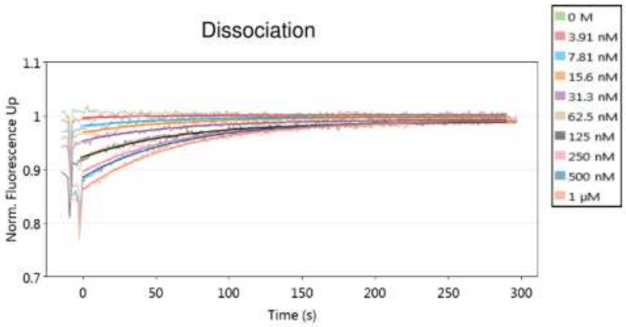

k_{ON} = 8.74 ± 0.36 E+6 $M^{-1} \cdot s^{-1}$

K_D = 1.72 ± 0.07 nM

k_{OFF} = 1.50 ± 0.01 E-2 s^{-1}

BAF S4E

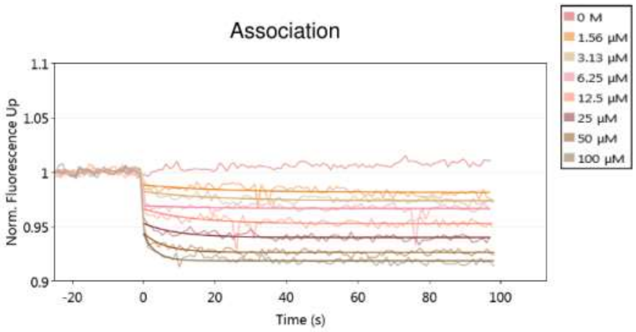

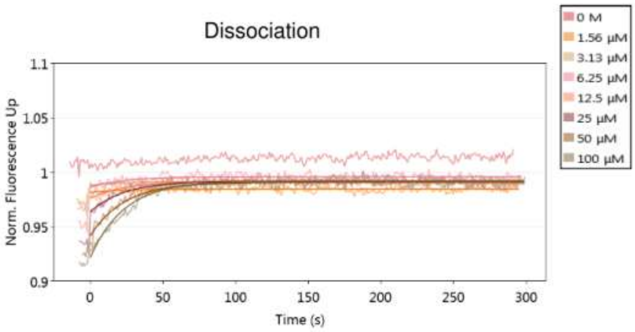

k_{ON} = 2.57 ± 0.44 E+3 $M^{-1} \cdot s^{-1}$

K_D = 16.7 ± 2.9 μM

k_{OFF} = 4.28 ± 0.08 E-2 s^{-1}

Suppl. figure 5

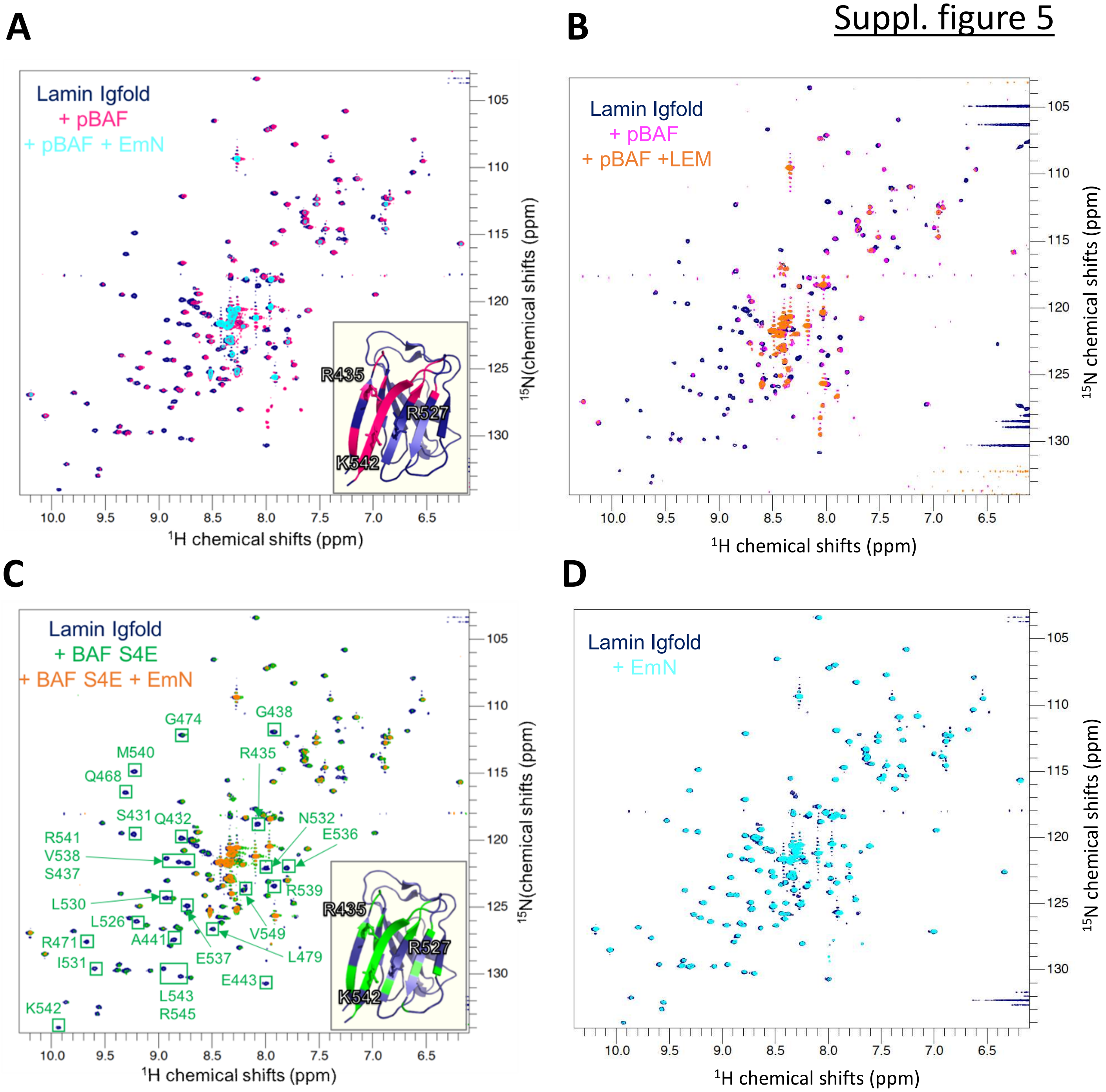

Suppl. Table 1

Data collection	pBAF	pBAF-Emerin
Wavelength (Å)	1.0332	0.8731
Space-group	P 32 2 1	P 1
Unit cells: a, b, c (Å), a, b, g (°)	70.32, 70.32, 40.63, 90.0, 90.0, 120.0	33.05, 40.6, 43.22, 69.5, 69.7, 85.0
Diffraction limits (last shell)	3.00 (3.18-3.00)	1.24 (4.00-1.24)
R-meas	0.196 (1.980)	0.163 (0.965)
CC(1/2)	0.998 (0.963)	0.989 (0.326)
I/s	7.8 (1.3)	4.5 (1.3)
Number of unique reflections	2463 (390)	32449 (1622)
Completeness (%)	100 (100)	84.8 (45.5)
Refinement		
Resolution (Å)	3.17	1.44
R/Rfree	0.196/0.326	0.183/0.230
RMSD bond length (Å)	0.0087	0.41
RMSD bond angles (°)	0.97	0.54
PDB entry code	7ABM	7DNY

R-meas = redundancy independent R-factor (intensities)
CC(1/2) = percentage of correlation between intensities from random half-datasets
I/σ = mean of intensity/Sigma(I) of unique reflections (after merging symmetry-related observations)

Suppl. Table 2

<table>
<tr><th></th><th>Kd (µM)</th><th>ΔH (cal/mol)</th><th>T*ΔS (cal/mol)</th><th>N</th><th>Kd (µM)</th><th>ΔH (cal/mol)</th><th>T*ΔS (cal/mol)</th><th>N</th></tr>
<tr><td>BAF vs LamIgF - 1</td><td>4.5 +/- 0.5</td><td>-33700 +/- 2500</td><td>-26035</td><td>0.4</td><td rowspan="2">4.5 +/- 0.6</td><td rowspan="2">-33650 +/-3300</td><td rowspan="2">-25978</td><td rowspan="2">0.6</td></tr>
<tr><td>BAF vs LamIgF - 2</td><td>4.4 +/- 0.6</td><td>-33600 +/-3300</td><td>-25920</td><td>0.8</td></tr>
<tr><td>pBAF vs LamIgF - 1</td><td>4.9 +/- 0.8</td><td>-7700 +/- 1400</td><td>-720</td><td>0.2</td><td rowspan="2">6.3 +/- 3.4</td><td rowspan="2">6500 +/- 1500</td><td rowspan="2">389</td><td rowspan="2">0.3</td></tr>
<tr><td>pBAF vs LamIgF - 2</td><td>7.7 +/- 3.4</td><td>-5300 +/- 1500</td><td>1498</td><td>0.4</td></tr>
<tr><td>BAF^{met} vs LamIgF</td><td>3.30 +/- 0.4</td><td>-17500 +/- 1000</td><td>-10483</td><td>0.4</td><td></td><td></td><td></td><td></td></tr>
<tr><td>pBAF^{met} vs LamIgF</td><td>3.60 +/- 0.7</td><td>-5300 +/- 500</td><td>1901</td><td>0.5</td><td></td><td></td><td></td><td></td></tr>
<tr><td>BAF vs LEM - 1</td><td>1.1 +/- 0.3</td><td>-8860 +/- 550</td><td>-1037</td><td>0.4</td><td rowspan="2">1.2 +/- 0.5</td><td rowspan="2">-8191 +/- 731</td><td rowspan="2">-389</td><td rowspan="2">0.4</td></tr>
<tr><td>BAF vs LEM - 2</td><td>1.3 +/- 0.6</td><td>-7521 +/- 731</td><td>259</td><td>0.4</td></tr>
<tr><td>pBAF vs LEM - 1</td><td>0.7 +/- 0.2</td><td>-2513 +/- 152</td><td>5587</td><td>0.3</td><td rowspan="2">0.9 +/- 0.4</td><td rowspan="2">-2462 +/- 204</td><td rowspan="2">5515</td><td rowspan="2">0.3</td></tr>
<tr><td>pBAF vs LEM - 2</td><td>1.1 +/- 0.4</td><td>-2411 +/- 204</td><td>5443</td><td>0.3</td></tr>
</table>
