# Supplementary material for: Di-phosphorylated BAF shows altered structural dynamics and binding to DNA, but interacts with its nuclear envelope partners
Source: Nucleic Acids Res. 2021 Mar 21;49(7):3841–55. doi: 10.1093/nar/gkab184 (PMC8053085; doi:10.1093/nar/gkab184)
Supplement: gkab184_Supplemental_Files [file gkab184_supplemental_files.zip › SupplFigures-Tables-REVISED-5feb-accepted.docx]

**Supplementary Figure 1. Phosphorylation of BAF by VRK1 characterized by SDS-PAGE and fluorescence. (A) SDS-PAGE experiment performed on BAF and pBAF.** In this gel, line 1 corresponds to the molecular weight markers, line 2 to a mix of VRK1 and BAF after 4h at 30°C, line 3 to purified pBAF and line 4 to BAF. **(B) Thermostability of pBAF, as measured using a Fluorescence-based Thermal Shift Assay.** A SYPRO Orange dye was mixed to the protein, and the fluorescence of the dye was followed with temperature on a QuantStudio 12KFlex qPCR machine (Applied Biosystems). Values obtained on BAF using a similar experimental setting are indicated for comparison.

**Supplementary Figure 2. MS analysis of BAF and pBAF. (A) Mass measurement of BAF and pBAF obtained by MALDI-TOF MS in linear mode.** MALDI-TOF experiments indicated that the mass difference between BAF (top) and pBAF (bottom) is 162 ± 2 Da. The error bars were calculated from 10 different MALDI-TOF experiments. Peaks corresponding to double (8476.8) and single (16952.6) charged ions of myoglobin were used as internal references. **(B) Phosphopeptide sequencing by MALDI-TOFTOF of the N-ter fragment of pBAF (obtained after EndoGlu-C digestion).** The zoom view highlights the pBAF_1-28_ fragments obtained after the first fragmentation step, leading to the conversion of the pSer and pThr into dehydroAla and methyldehydroAla by a β-elimination reaction (also shown in Figure 3B; (51,52)). MSMS sequencing further provided the mass of the Y_x_ peptides, x corresponding to the number of amino acids of the N-terminally truncated peptides: for example, Y_27_ is fragment 2-28, Y_26_ is fragment 3-28, Y_25_ is fragment 4-28. B_x_ are the C-terminally truncated peptides, which were not used for this analysis. The position of the phosphorylated residues was deduced from the identification of dehydroAla and methyldehydroAla residue-containing ions (star labels). For comparison, the same analysis was performed on BAF_1-28_ (data not shown).

**Supplementary Figure 3. Real-time NMR monitoring of the phosphorylation of BAF WT and mutants**. 2D NMR ^1^H-^15^N HSQC spectra were recorded every 30 min after addition of VRK1 at pH 7.2, 30°C and 700 MHz. **(A) Superimposition of the spectra acquired on BAF WT at 0 (grey), 0.5 (blue) and 2 (pink) hours.** Peaks identified as strongly shifting in Figures 2A and 4A are marked with either boxes or arrows. **(B) Superimposition of the initial and final states of the phosphorylation kinetics of BAF T3A and S4E by VRK1.** Here again, the detectable signals previously identified as strongly shifting due to BAF phosphorylation are surrounded by dotted boxes (in the case of pBAF S4E, the weak signal corresponding to G16 is displayed as a green cross, and signals corresponding to V11 and E13 could not be unambiguously assigned; in the case of pBAF T3A and S4E, the signal of the side chain of W84 is not detected). Zoom views of the glycine region are presented. In the case of T3A, this view includes the spectrum of the mono-phosphorylated BAF WT species (in blue as in (A)). In the case of S4E, it includes the spectrum of the di-phosphorylated BAF WT species (in pink as in (A)). The peaks boxed in cyan might correspond to a minor species phosphorylated on Thr2, which is not observed in the case of BAF WT and BAF S4E.

**Supplementary Figure 4. Representative fluorescence experiments that monitor binding of BAF WT, A12T and S4E to a coated 48 nt dsDNA revealed that the phosphomimetic BAF S4E, but not the NGPS-associated BAF A12T, exhibits a significantly decreased affinity for dsDNA compared to BAF WT.** Affinities calculated from all fluorescence experiments are reported in **Table 1**.

**Supplementary Figure 5. NMR monitoring of ^15^N-labeled LamIgF binding to either pBAF and EmN, pBAF and LEM, BAF S4E and EmN or only EmN. (A) Interaction between LamIgF and pBAF either free or bound to the emerin nucleoplasmic region EmN.** 2D NMR ^1^H-^15^N HSQC spectra were recorded on ^15^N-labeled LamIgF either free (dark blue), in the presence of a 1:1 molecular ratio of BAF (pink; the molecular ratio corresponds to 1 LamIgF for 0.5 pBAF dimer) or in the presence of a 1:1:1 molecular ratio of pBAF and EmN (cyan). A 3D view of the lamin Igfold is inserted (PDB 6GHD), highlighting the spatial distribution of the residues whose HSQC peak intensity decreases by at least 80% after addition of pBAF (in pink). The side chains of Arg435, Arg527 and Lys542, whose mutations affect binding to BAF (28), are displayed in sticks and marked. **(B) Interaction between LamIgF and pBAF either free or bound to the LEM domain of emerin.** 2D NMR ^1^H-^15^N HSQC spectra were recorded on ^15^N-labeled LamIgF either free (dark blue), in the presence of a 1:1 molecular ratio of pBAF (pink; the molecular ratio corresponds to 1 LamIgF for 0.5 pBAF dimer) or in the presence of a 1:1:1 molecular ratio of pBAF and LEM (orange). After addition of pBAF and then EmN or LEM, only peaks corresponding to the unfolded region of LamIgF are observed. **(C) Interaction between LamIgF and BAF S4E either free or bound to EmN.** 2D NMR ^1^H-^15^N HSQC spectra were recorded on ^15^N-labeled LamIgF either free (dark blue), in the presence of a 1:1 molecular ratio of BAF S4E (green; the molecular ratio corresponds to 1 LamIgF for 0.5 BAF S4E dimer) or in the presence of a 1:1:1 molecular ratio of BAF S4E and EmN (orange). Peaks whose intensity decreases by at least 80% after addition of BAF S4E are boxed and labeled. A 3D view of the lamin Igfold is inserted (PDB 6GHD), highlighting the spatial distribution of the residues corresponding to these peaks (in green). Here again, after addition of EmN, only peaks corresponding to the unfolded region of LamIgF are observed. **(D) Control experiment testing direct binding of LamIgF to EmN.** 2D NMR ^1^H-^15^N HSQC spectra were recorded on ^15^N-labeled LamIgF either free (dark blue) or in the presence of a 1:1 molecular ratio of EmN (cyan). All experiments were recorded at 20°C and 700 MHz.

**Suppl. Table 1. Data collection and refinement statistics** (PDB entries 7ABM and 7DNY).

**Suppl. Table 2. Summary of the affinities, stoichiometries, enthalpies and entropies obtained from two ITC experiments performed between BAF or pBAF and LamIgF, one ITC experiment performed between BAF_met_ or pBAF_met_ and LamIgF, and two ITC experiments performed between BAF or pBAF and LEM.** BAF_met_ exhibits a native Met1 residue instead of the Gly1 present in the construct named BAF.
